# Haplotyping by linked-read sequencing (HLRS) of the genetic disease carriers for preimplantation genetic testing without a proband or relatives

**DOI:** 10.1186/s12920-020-00766-1

**Published:** 2020-08-20

**Authors:** Qing Li, Yan Mao, Shaoying Li, Hongzi Du, Wenzhi He, Jianchun He, Lingyin Kong, Jun Zhang, Bo Liang, Jianqiao Liu

**Affiliations:** 1 The Third Affiliated Hospital of Guangzhou Medical College, 63 Duobao Road, Guangzhou, 510150 Guangdong Province China; 2 Basecare Medical Device Co., Ltd, 218 Xinghu Road, Suzhou, 215001 Jiangsu Province China; 3 School of Life Sciences and Biotechnology, Shanghai Jiao Tong University, 800 Dongchuan Road, Shanghai, 200240 China

**Keywords:** Preimplantation Genetic Testing for monogenic disease, Linked-read sequencing, Linkage analyses, Haplotype

## Abstract

**Background:**

In order to mitigate the risk of allele dropout (ADO) and ensure the accuracy of preimplantation genetic testing for monogenic disease (PGT-M), it is necessary to construct parental haplotypes. Typically, haplotype resolution is obtained by genotyping multiple polymorphic markers in both parents and a proband or a relative. Sometimes, single sperm typing, or tests on the polar bodies may also be useful. Nevertheless, this process is time-consuming. At present, there was no simple linkage analysis strategy for patients without affected relatives.

**Method:**

To solve this problem, we established a haplotyping by linked-read sequencing (HLRS) method without the requirement for additional relatives. First, the haplotype of the genetic disease carriers in the family was constructed by linked-read sequencing, and then the informative single nucleotide polymorphisms (SNPs) in upstream and downstream mutation region were selected to construct the embryo haplotype and to determine whether the embryo was carrying the mutation. Two families were selected to validate this method; one with alpha thalassemia and the other with NDP gene disorder.

**Results:**

The haplotyping by linked-read sequencing (HLRS) method was successfully applied to construct parental haplotypes without recruiting additional family members; the method was also validated for PGT-M. The mutation carriers in these families were sequenced by linked-read sequencing, and their haplotypes were successfully phased. Adjacent SNPs of the mutation gene were identified. The informative SNPs were chosen for linkage analyses to identify the carrier embryos. For the alpha thalassemia family, a normal blastocyst was transferred to the uterus and the accuracy of PGT-M was confirmed by amniocentesis at 16 weeks of gestation.

**Conclusions:**

Our results suggest that HLRS can be applied for PGT-M of monogenic disorders or de novo mutations where the mutations haplotype cannot be determined due to absence of affected relatives.

## Background

In order to reduce birth defects, couples with genetic disorders are heavily reliant on modern reproductive medicine. Preimplantation genetic testing for monogenic disease (PGT-M) is a clinical diagnostic procedure that can effectively prevent the implantation of embryos with genetic defects, without the need of gamete donation or adoption [1, 2]. Since early 1990s, PGT-M has been applied in various genetic disorder cases, from X-linked genetic diseases to multiple monogenic disorders [3, 4].

The most widely used techniques for PGT-M generally rely on single-cell polymerase chain reaction (PCR) amplification of the target genes. Nevertheless, due to the unavoidable allele dropout (ADO), direct PCR approach for detection of the target pathogenic mutation sites cannot be used as the sole method for diagnosis [5]. ADO refers to the failure of one of the two alleles of a heterozygous locus. This makes a heterozygous cell appear homozygous at the affected locus, leading to misdiagnosis. Over recent years, linkage analysis method has been widely used to increase PGT-M accuracy [6]. The affected and unaffected embryos are distinguished using haplotype-phasing results, where the familial linkage analyses are performed using both the couple’s and a proband’s or relatives’ genome.

Linkage analysis relies on single nucleotide polymorphism (SNP) or short tandem repeat (STR) markers linked to the mutations. In pre-examination process, SNP or STR marker genotyping is performed on DNA samples of the couple and related family members to identify informative markers and to establish the couple’s haplotype. The informative markers are then detected in embryos to confirm the haplotype of the embryos and to determine whether the embryos carry familial mutations. However, under certain conditions, such as inclusion of cases with de novo mutations or lack of familial samples, haplotypes cannot be phased using classical approaches in preimplantation genetic haplotyping. Under such conditions, phasing haplotypes in single sample without familial analysis is a potential alternative approach.

In this study, we presented a method called haplotyping with linked-read sequencing (HLRS), which can be used to construct the carrier’s haplotype without including the family members. Two carrier families were selected to validate this method, one with alpha thalassemia and one with Norrie disease. These two families were chosen as representatives of two main types of genetic disorders: gene deletion and point mutation. This study demonstrated the feasibility of using HLRS technology to PGT-M for different diseases and provided a new solution for clinical application of PGT-M.

## Methods

### Patients

Two families were selected at the Third Affiliated Hospital of Guangzhou Medical University; and the informed consents were obtained from patients. The wife (sample G2018004A_LYL) and husband (sample G2018004B_LGB) from family one were both alpha thalassemia Southeast Asian (*αα/−−SE*A) deletion carriers. The wife (sample G2018001A_LSL) from second family was a carrier of a heterozygous point mutation(*c.122G > A*)in exon 2 of the NDP gene and her husband was normal. 1-ml of peripheral blood from each sample was collected in EDTA anticoagulant tube for genomic DNA (gDNA) preparation.

### Sample preparation

Genomic DNA was extracted using MagAttract HMW DNA Kit (QIAGEN, Hilden, Germany). After gDNA extraction, product length, quantity and purity were checked by Nanodrop spectrometry (Thermo Fisher Scientific, Waltham, MA, USA) and pulsed-field gel electrophoresis (PFGE; 1%AGE, 6 V, 18 h). Qualified DNAs were expected to reach following values: (1) conc. > 20 ng/μl; (2) OD260/280 between 1.8–2.0; (3) OD260/230 between 2.0–2.2; (4) total amount extracted > 1.5 μg; (5) main band in PFGE > 50 Kb and; (6) no detectable protein contamination.

### HLRS procedure

**Fig. 1:**
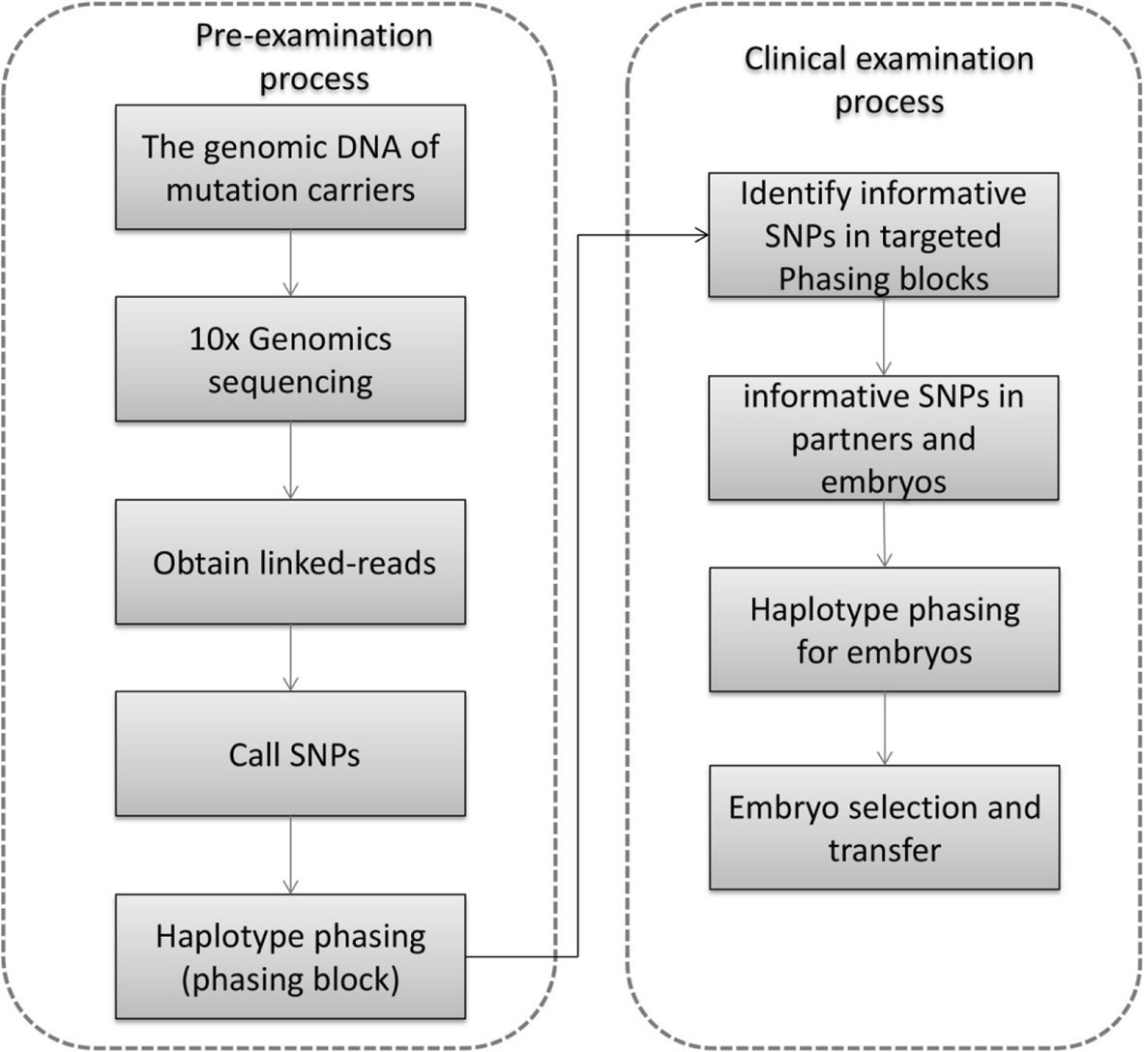
The workflow of HLRS for PGT-M. Black arrows show the flow of information from the samples to the final identification results. The dotted frame on the left shows the pre-examination process to construct the carrier’s haplotype; the dotted frame on the right shows the clinical examination process to test the embryos

HLRS for PGT-M included two processes, the pre-examination process and clinical examination process (Fig. 1). In pre-examination process, 10× Genomics was used to perform linked-read sequencing and whole genome haplotyping. During clinical examination process, the informative SNPs were selected and used to phase the embryo’s haplotype. Finally, the normal embryo was selected for implantation.

### Linked-read sequencing and whole genome haplotyping

10× Genomics was used to do linked-read sequencing and whole genome haplotyping. Barcoding and library construction were performed using 10× Chromium (10× Genomics, Pleasanton, California, USA). After checking quality, the library was sequenced using Illumina HiSeq PE150. Sequencing data were processed using 10× Analysis Suite (10× Genomics). After evaluating data quality and standard preprocessing, Lariat algorithm and FreeBayes algorithm of Long Ranger software developed by 10× Genomics were used to map linked-reads to reference genome and call SNPs. Phasing of the linked-reads was performed using the Long Ranger software where single base variants in linked-reads are strung together into haplotype blocks [7].

### SNP selection for linkage analysis

The carriers’ haplotypes were established using the phase blocks containing the pathogenic mutation. The SNPs flanking the mutation in targeted phase block were picked as candidate SNPs. Only the SNPs that were heterozygous in the carriers and homozygous in their partner were considered as informative SNPs. We synthesized specific primers for amplification and then sequenced the selected SNPs of the couples.

### Embryo diagnosis

All oocytes from these two couples were fertilized by intracytoplasmic sperm injection (ICSI) and cultured following a standard blastocyst culture procedure. Approximately three to five trophectoderm cells were biopsied from each blastocyst on day 5. PicoPLEX Single Cell WGA Kit (Takara Bio, Dalian, China) was used for whole genome amplification (WGA) of the biopsied trophectoderm cells. The informative SNPs flanking the mutation were amplified via PCR in the WGA products and then sequenced. The embryos’ haplotypes were phased using these SNPs.

### Embryo transfer

A single unaffected blastocyst was transferred to the uterus in both families. Clinical pregnancy was confirmed when an intrauterine gestational sac with a heartbeat was observed via ultrasound examination 30–40 days after embryo transfer. Amniocentesis was performed at 16 weeks’ gestation age. The amniocentesis fluid samples from fetuses were used for prenatal diagnosis to confirm the PGT-M results.

## Results

### Profiles of 10× genomics sequencing results

According to previous studies [8, 9], with sequencing depth of under around 35× (120 GB raw sequencing data), N50 phase block can be over 500 kb. In this study, three carrier samples were tested. All samples except G2018001A_LSL had a sequencing depth of more than 35×, and all samples had an N50 phase block of more than 500 kb. As summarized in Table 1, the sequencing depth of each sample was 47.4×, 40.0× and 33.5× respectively. After phasing analysis, 95.9, 94.9 and 95.6% SNPs were phased. The longest phase block could reach 5,080,496 bp, 5,421,595 bp and 3,620,840 bp, with N50 phase block of 775,060 bp, 840,402 bp and 684,981 bp respectively.

**Table 1:** 10× Genomics sequencing summary

Sample	G2018004A_LYL	G2018004B_LGB	G2018001A_LSL
Input DNA Molecular Length	21,858 bp	24,088 bp	22,631 bp
Number of Reads	1091.38 M	937.20 M	776.89 M
Mapped Reads	95.9%	94.9%	95.6%
Median Insert Size	365 bp	360 bp	363 bp
Mean Depth	47.4×	40.0×	33.5×
SNPs Phased	99.2%	98.4%	98.9%
Longest Phase Block	5,080,496 bp	5,421,595 bp	3,620,840 bp
N50 Phase Block	775,060 bp	840,402 bp	684,981 bp

*SNPs* Single nucleotide polymorphisms

### Haplotype phase blocks

**Fig. 2:**
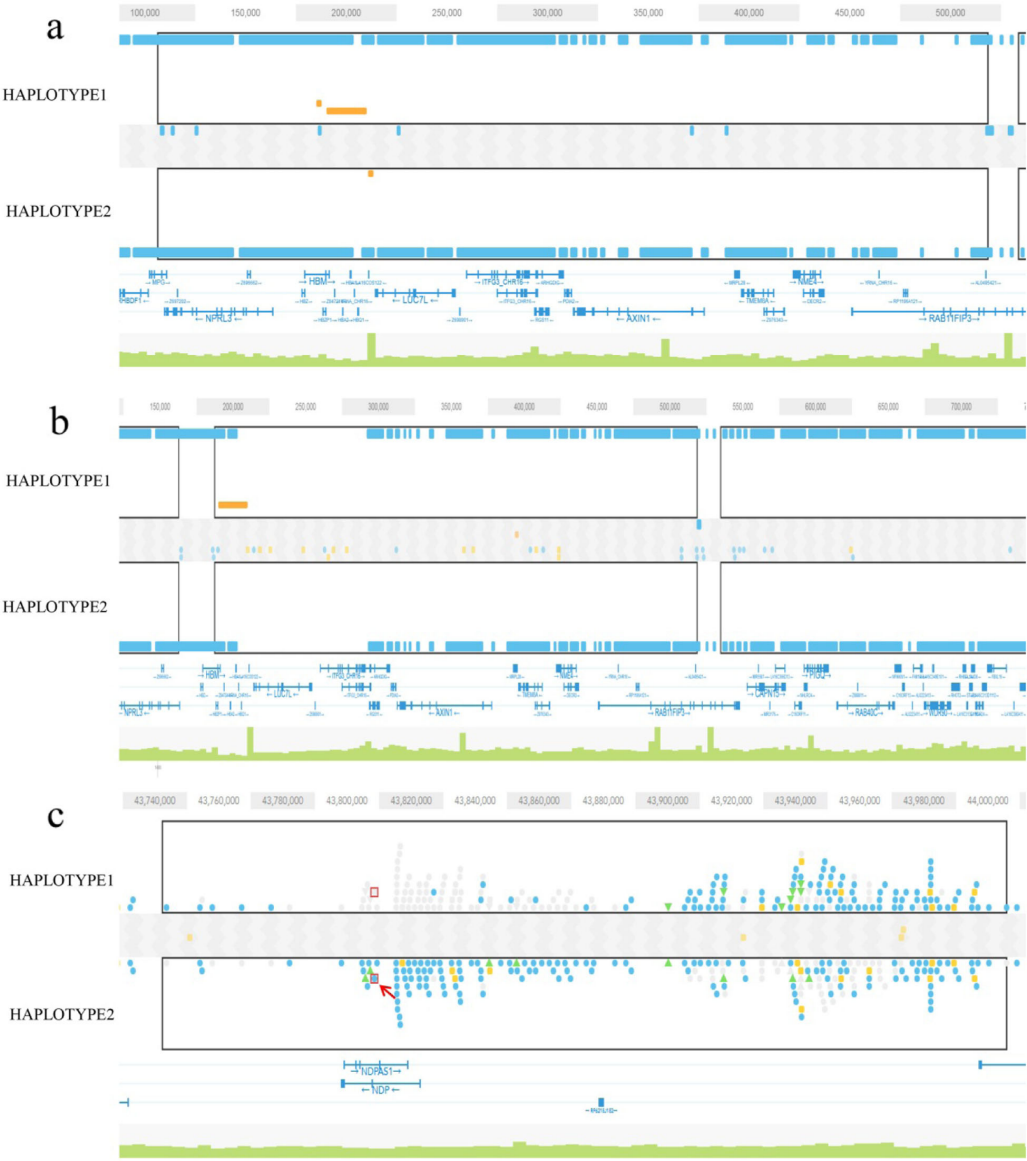
Targeted phase blocks in three samples. **a** G2018004A_LYL; **b** G2018004B_LGB; **c** G2018001A_LSL. The black box represents the phase block in which the mutant gene is located, the orange line represents the HBA gene deletion region, and the position indicated by the arrow represents NDP c.122G > A

Targeted phasing regions for respective pathogenic mutation sites in each sample were all covered by phase blocks (Table 2). For alpha thalassemia carrier samples, the corresponding phase block for G2018004A_LYL was 410.kb long and contained 309 heterozygous SNPs, while that for G2018004B_LGB was only 319.4 kb with 251 heterozygous SNPs. For NDP disorder carrier sample G2018001A_LSL, the corresponding phase block was 262.3 kb long with 134 heterozygous SNPs. The carrier allele and normal allele could also be distinguished using these phase blocks (Fig. 2). For alpha thalassemia carrier samples, there was a HBA gene deletion in haplotype 1, which means haplotype 1 was the carrier allele. For NDP disorder carrier sample, there was a NDP gene mutation (c.122G > A) in haplotype 2, which means haplotype 2 was the carrier allele.

**Table 2:** Targeted Phasing Results Summary

Sample	Chr	Targeted Phasing Region	Belonged Phase block	Phase Block Length	Contained Heterozygous SNPs
G2018004A_LYL	16	215,400–234,700	132,167–542,445	410.2 kb	309
G2018004B_LGB	16	215,400–234,700	223,804–543,224	319.4 kb	251
G2018001A_LSL	X	43,817,770	43,752,981–44,015,286	262.3 kb	134

*SNPs* Single nucleotide polymorphisms

Targeted phasing region, the pathogenic mutation sites for respective samples; Belonged Phase block, the phase block that contained the mutation sites

### Informative SNPs selection

The SNPs that were heterozygous in the carriers and homozygous in their partner were considered as informative SNPs. Based on the sequence information obtained for the flanking mutations, we identified 436 informative SNPs in family 1. Among them, 286 SNPs in the target phase block were heterozygous in the wife and homozygous in the husband; conversely, 150 SNPs were heterozygous in the husband and homozygous in the wife. In family 2, 134 SNPs were heterozygous in the wife. We selected 10 informative SNPs in each family to test their embryos.

### PGT-M

A total of 10 blastocysts were biopsied for WGA; 5 for each family. Biopsy trophectoderm cells from 10 blastocysts were successfully amplified. Next, PCR analysis that spanned the mutations was performed. In total, 10 SNPs within phase block of the mutations were successfully analyzed in each family. Next, the linkage analysis with the informative SNPs in 10 embryos of two families was performed. The linkage analysis showed that Embryo1–1, Embryo1–2, Embryo1–5 (Table 3) Embryo2–3, and Embryo2–4 (Table 4**)** inherited the normal haplotypes. However, Embryo1–3, Embryo2–1, Embryo2–2 and Embryo2–5 inherited an affected haplotype from the mother (solid square box), and Embryo1–4 inherited affected haplotypes from the parents (solid square box and dotted square box).

**Table 3:**
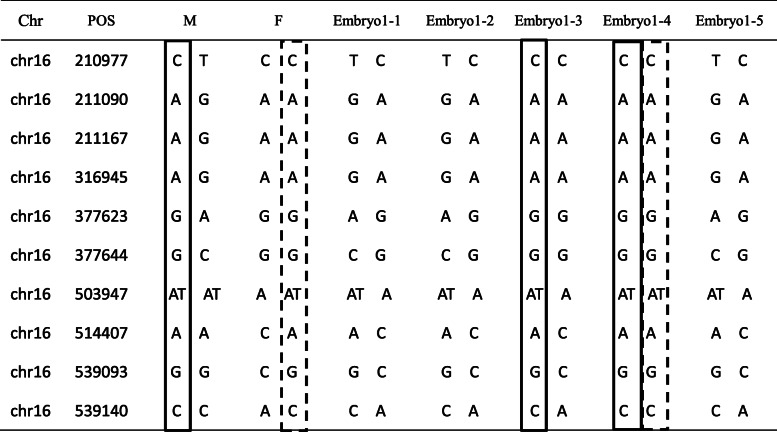
Ten SNP markers were selected to construct the haplotypes of embryos in family 1

*POS* position, *M* Mother, *F* Father

solid square box, affected haplotype from the mother; dotted square box, affected haplotype from the father

**Table 4:**
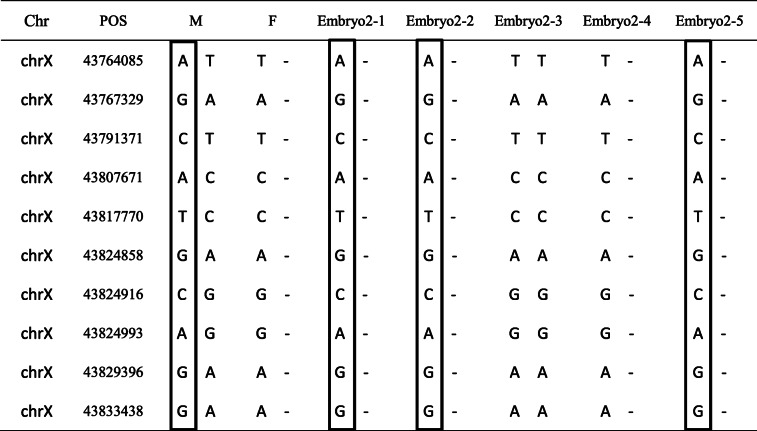
Ten SNP markers were selected to construct the haplotypes of embryos in family 2

*POS* position, *M* Mother, *F* Father

solid square box, affected haplotype from the mother

### Clinical outcome

Embryo1–2 and Embryo2–3 were transferred to their mothers’ uterus, resulting in pregnancy in both families. Unfortunately, an unexplained abortion occurred in family 1 during the first trimester. However, amniocentesis was performed in family 2 at 16 weeks of gestation to verify the accuracy of the PGT-M. After 38 weeks of pregnancy, the family 2 got a healthy baby girl.

## Discussion

PGT-M is an alternative approach to prenatal diagnosis for the detection of genetic disorders in couples at risk of transmitting a genetic condition to their offspring. PCR-based methods have been used for PGT-M over the past decades. Yet, all single-cell based PCR tests share a common problematic phenomenon known as ADO. ADO is one of the main causes of misdiagnosis in PGT-M, especially for dominant disorders [10, 11]. Linkage analysis of polymorphic markers and mutated genes was used to establish a haplotyping method for identifying ADO and ensuring the accuracy of PGT-M. In previous studies, family samples were usually used for linkage analysis to construct haplotypes for both husband and wife. Nonetheless, in some specific cases, such as certain severe genetic disorders, when the mutation-carrying proband is deceased, when the couple does not know to be the carrier of hereditary diseases and never had children, or when informative relatives are not available, the necessary family samples were missing.

Over the past few years, establishing the haplotypes through single sperm and polar bodies when lacking related relatives or in cases of de novo mutations has been a recommended approach. Wu et al presented successful PGD procedure for a couple carrying HBB mutations based on genotyping multiple single-sperm cells to obtain SNP linkage information [12]. Furthermore, Chen et al used single sperm and applied next-generation sequencing (NGS) to analyze the genotypes of the SNP alleles for the PGT-M of COL1A1 mutation [13]. But it may be more complex in the sperms and polar bodies analysis. It requires an extra biopsy produce in the polar bodies’ extracting and often needs multiple single-sperms to establish the male’s haplotypes.

Long read sequencing of the mutation specific amplicons from the carriers is another strategy to establish the haplotypes that has been developed over recent years. This approach is based on two key requirements: a long sequence length, where the minimal requirement is several kilo bases necessary to cover at least two heterozygous SNPs so that different detected sequences could overlap and extend to make a usable phase block. The other requirement is single nucleotide precision, which is indispensable for identifying pathogenic mutation sites and phasing haplotypes using SNPs as biomarkers. 10× Genomics sequencing technologies that use unique linked-reads based library preparation method meet both of these two requirements, and have already been proven to have the ability of genome-wide haplotype phasing in previous pilot studies [14–16].

In this study, we applied HLRS method based on 10× Genomics sequencing to successfully phase haplotypes in samples from patients who were carriers of alpha thalassemia or NDP gene disorder. After successful haplotype phasing of these patient samples, we identified these SNPs in patients’ embryos and then phased the haplotypes of their embryos. In each patient, a healthy embryo without inherited monogenic disorders was implanted resulting in a healthy baby in family 2. During the embryos phasing, we used Sanger sequencing to identify these SNPs. However, when there are many SNPs or embryos, this method is laborious and tedious. Maybe multiplex PCR amplification combined with high-throughput sequencing are suitable.

HLRS method resolved the limitations of other methods: easy to perform, rich SNPs, and no need for familial samples. The successful construction of the haplotype of the mutated region is essential for application of this method. However, due to the randomness mainly introduced in library preparation of 10× Genomics sequencing method, the success rate of phasing certain pathogenic mutation site cannot be 100% guaranteed, especially in some complex regions of the genome. In the future, we will expand this method on some families with mutations at different genomic locations, such as highly repetitive regions, high GC content regions, or variant deserts. Whether the target phasing region of a particular case could be successfully phased or not also depends on the quality of input DNA and sequencing depths. Both longer input DNA fragments and higher DNA sequencing depth help to generate better phasing results. In addition, the cost of 10x genomics technology is relatively exorbitant. In clinical, it is only suitable for PGT- M cases where mutation carrier family members are not available to enable parent haplotype phasing. It is not recommended to families with an affected proband.

## Conclusions

In this study, we applied HLRS method to successfully phase haplotypes in samples from patients who were carriers of alpha thalassemia or NDP gene disorder, without including their family members. This study demonstrated the feasibility of using HLRS method in PGT-M for monogenic disorders or de novo mutations where the mutations haplotype cannot be determined due to absence of affected relatives, and provided a new solution for clinical research of PGT-M. HLRS is a promising PGT-M strategy that can make up for the shortcomings of the current technology, even though only few cases were tested in the current study. In the future, the feasibility of this method should be verified with larger sample sizes.

## Data Availability

The datasets generated and analyzed during the current study are not publicly available due to a concern to protect individual patient confidentiality but are available from the corresponding author on reasonable request.

## Abbreviations

HLRS: Haplotyping by linked-read sequencing
ADO: Allele dropout
PGT-M: Preimplantation genetic testing for monogenic disease
SNPs: Single nucleotide polymorphisms
STR: Short tandem repeat
ICSI: Intracytoplasmic sperm injection
WGA: whole genome amplification

## References

[1] Sermon K, Van Steirteghem A, Liebaers I (2003). Preimplantation genetic diagnosis. Lancet.

[2] Ogilvie CM, Braude P, Scriven PN (2005). Preimplantation genetic diagnosis—an overview. J Histochem Cytochem.

[3] Handyside AH, Lesko J, Tarin JJ, Winston R, Hughes MR (1992). Birth of a Normal girl after in vitro fertilization and preimplantation diagnostic testing for cystic fibrosis. N Engl J Med.

[4] Handyside AH, Kontogianni EH, Hardy K, Winston R (1990). Pregnancies from biopsied human preimplantation embryos sexed by Y-specific DNA amplification. Nature..

[5] Harton G, De Rycke M, Fiorentino F, Moutou C, Sengupta S, Traegersynodinos J (2011). ESHRE PGD consortium best practice guidelines for amplification-based PGD. Hum Reprod.

[6] Thornhill AR, Handyside AH, Ottolini CS, Natesan SA, Taylor J, Sage K (2015). Karyomapping—a comprehensive means of simultaneous monogenic and cytogenetic PGD: comparison with standard approaches in real time for Marfan syndrome. J Assist Reprod Genet.

[7] Mostovoy Y, Levysakin M, Lam J, Lam ET, Hastie A, Marks P (2016). A hybrid approach for de novo human genome sequence assembly and phasing. Nat Methods.

[8] Hui WWI, Jiang P, Tong YK, Lee W, Cheng YKY, New MI (2017). Universal haplotype-based noninvasive prenatal testing for single gene diseases. Clin Chem.

[9] Zheng GXY, Lau BT, Schnalllevin M, Jarosz M, Bell JM, Hindson C (2016). Haplotyping germline and cancer genomes with high-throughput linked-read sequencing. Nat Biotechnol.

[10] Wilton L, Thornhill AR, Traegersynodinos J, Sermon K, Harper JC (2009). The causes of misdiagnosis and adverse outcomes in PGD. Hum Reprod.

[11] Harper JC, Wilton L, Traegersynodinos J, Goossens V, Moutou C, Sengupta S (2012). The ESHRE PGD consortium: 10 years of data collection. Hum Reprod Update.

[12] Wu H, Shen X, Huang L, Zeng Y, Gao Y, Shao L (2018). Genotyping single-sperm cells by universal MARSALA enables the acquisition of linkage information for combined pre-implantation genetic diagnosis and genome screening. J Assist Reprod Genet.

[13] Chen L, Diao Z, Xu Z, Zhou J, Yan G, Sun H (2019). The clinical application of single-sperm-based SNP haplotyping for PGD of osteogenesis imperfecta. Syst Biol Reprod Med.

[14] Jang SS, Lim BC, Yoo SK, Shin JY, Kim KJ, Seo J (2018). Targeted linked-read sequencing for direct haplotype phasing of maternal DMD alleles: a practical and reliable method for noninvasive prenatal diagnosis. Sci Rep.

[15] Zhou X, Batzoglou S, Sidow A, Zhang L (2018). HAPDeNovo: a haplotype-based approach for filtering and phasing de novo mutations in linked read sequencing data. BMC Genomics.

[16] Kitzman JO (2016). Haplotypes drop by drop. Nat Biotechnol.

